# Complex Upper-Limb Movements Are Generated by Combining Motor Primitives that Scale with the Movement Size

**DOI:** 10.1038/s41598-018-29470-y

**Published:** 2018-08-27

**Authors:** Jose Garcia Vivas Miranda, Jean-François Daneault, Gloria Vergara-Diaz, Ângelo Frederico Souza de Oliveira e Torres, Ana Paula Quixadá, Marcus de Lemos Fonseca, João Paulo Bomfim Cruz Vieira, Vitor Sotero dos Santos, Thiago Cruz da Figueiredo, Elen Beatriz Pinto, Norberto Peña, Paolo Bonato

**Affiliations:** 1 Institute of Physics, Laboratory of Biosystems, Universidade Federal da Bahia, Salvador, BA Brazil; 2 Department of Physical Medicine and Rehabilitation, Harvard Medical School, Spaulding Rehabilitation Hospital, Boston, MA USA; 3 Faculdade Social da Bahia, Salvador, BA Brazil; 4 Institute of Medical Psychology and Behavioural Neurobiology, Eberhard Karls University of Tübingen, Tübingen, Germany; 5 Motor Behavior and Neurorehabilitation Research Group, Bahiana School of Medicine and Public Health, Salvador, BA Brazil; 6 Wyss Institute for Biologically Inspired Engineering, Harvard University, Boston, MA USA

## Abstract

The hand trajectory of motion during the performance of one-dimensional point-to-point movements has been shown to be marked by motor primitives with a bell-shaped velocity profile. Researchers have investigated if motor primitives with the same shape mark also complex upper-limb movements. They have done so by analyzing the magnitude of the hand trajectory velocity vector. This approach has failed to identify motor primitives with a bell-shaped velocity profile as the basic elements underlying the generation of complex upper-limb movements. In this study, we examined upper-limb movements by analyzing instead the movement components defined according to a Cartesian coordinate system with axes oriented in the medio-lateral, antero-posterior, and vertical directions. To our surprise, we found out that a broad set of complex upper-limb movements can be modeled as a combination of motor primitives with a bell-shaped velocity profile defined according to the axes of the above-defined coordinate system. Most notably, we discovered that these motor primitives scale with the size of movement according to a power law. These results provide a novel key to the interpretation of brain and muscle synergy studies suggesting that human subjects use a scale-invariant encoding of movement patterns when performing upper-limb movements.

## Introduction

For over a century, researchers have attempted to identify simple principles that account for the generation of upper-limb movements in human subjects^1,2^. Observations gathered during the performance of motor tasks such as handwriting and drawing geometric shapes allowed Viviani and Terzuolo^3^ to conclude that the instantaneous velocity of curvilinear movements is proportional to the radius of curvature of the trajectory of movement. Subsequently, Lacquaniti *et al*.^4^ derived the following power law:

1 $${\rm{v}}({\rm{t}}){\tilde{=}k}_{{\rm{v}}}{r(t)}^{1/3}$$

where ${\rm{v}}(.)$ is the instantaneous velocity of movement, ${k}_{v}$ is a constant that depends on movement characteristics such as the size of the movement, $r(.)$ is the radius of curvature, and $t$ is time. Equation 1 is referred to as the “one-third power law”. Lacquaniti *et al*.^4^ also showed that the movement trajectory can be represented as the angle $\theta (t)$ between the tangent to the trajectory of movement and either of the axes of an arbitrarily set Cartesian reference system. It follows that the angular velocity $\frac{{\rm{d}}}{{\rm{dt}}}{\rm{\theta }}({\rm{t}})$ is a function of the curvature of the trajectory of movement $C(t)$:

2 $$\frac{{\rm{d}}}{{\rm{dt}}}{\rm{\theta }}(t)\tilde{=}{{k}}_{{\theta }}C{(}t{{)}}^{2/3}$$

where ${k}_{\theta }$ is a constant with characteristics similar to ${k}_{v}$. Further studies showed that Equations 1 and 2 hold in principle for a large class of curvilinear movements, though modifications of these equations were proposed for complex movements^5,6^. For instance, Huh and Sejnowski^7^ used a spectrum of power laws to model complex curvilinear movements.

Movements that satisfy the one-third power law satisfy the minimum-jerk principle^8–10^ proposed by Flash and Hogan^11^. Furthermore, curvilinear movements that satisfy the one-third power law are marked by constant affine velocity^12^ (i.e. the velocity of movement as described using affine geometry^13^- a non-Euclidean geometry). Simple principles underlying the control of movement can be identified using non-Euclidean transformations^14–16^ and such principles are consistent with the minimum-jerk principle^17–19^. However, this body of work neglects to consider the “cost of time”, a principle that has been suggested to play a key role in the control of movement^20–23^.

A model that accounts for the “cost of time” and that is consistent with the minimum-jerk principle was proposed by Hoff  ^24^ to study two-dimensional arm reaching movements based on the following cost function:

3 $${\rm{I}}={{\rm{t}}}_{{\rm{f}}}+{\rm{K}}{\int }_{{\rm{t}}=0}^{{\rm{t}}={{\rm{t}}}_{{\rm{f}}}}({{\rm{u}}}_{{\rm{x}}}^{2}+{{\rm{u}}}_{{\rm{y}}}^{2}){\rm{dt}}$$

where ${t}_{f}$ is the duration of the movement, ${u}_{x}$ and ${u}_{y}$ are the x- and y-axis components of the jerk time series, and $K$ is a constant. Hoff derived a polynomial expression of the trajectory of point-to-point movements that minimizes the cost function $I$. Assuming static boundary conditions, he then derived the relationship between the duration of the movement ${t}_{f}$ and the associated displacement $D$:

4 $${{\rm{t}}}_{{\rm{f}}}{=(\text{60D})}^{1/3}{{\rm{K}}}^{1/6}$$

Hoff also showed that one-dimensional point-to-point movements are marked by a velocity profile ${\rm{v}}({\rm{t}})$ that is approximately bell-shaped and obeys the following equation:

5 $${\rm{v}}({\rm{t}})={\rm{D}}[\frac{30}{{{{\rm{t}}}_{{\rm{f}}}}^{5}}{{\rm{t}}}^{4}-\frac{60}{{{{\rm{t}}}_{{\rm{f}}}}^{4}}{{\rm{t}}}^{3}+\frac{30}{{{{\rm{t}}}_{{\rm{f}}}}^{3}}{{\rm{t}}}^{2}]$$

It is worth emphasizing that Hoff developed this theoretical framework for two-dimensional arm-reaching movements.

As we sought to identify the principles underlying the generation of complex upper-limb movements, we carried out a series of experiments to explore if a broad category of upper-limb movements could be looked upon as a combination of one-dimensional point-to-point movements. In contrast to previous studies that focused on the analysis of the magnitude of the hand trajectory velocity vector^25–30^, we analyzed the hand trajectory components defined according to a Cartesian coordinate system with axes oriented according to the subject’s anatomical planes, i.e. with axes oriented in the medio-lateral, antero-posterior, and vertical direction, respectively. Specifically, we tested the hypothesis that the movement components along the axes of this coordinate system consist of a sequence of elements obeying Equation 5.

Furthermore, we observed that elements obeying Equation 5 also obey Equation 4 and hence they scale with the movement size according to the following equation relating the mean velocity of movement $\bar{v}$ and the associated displacement $D$:

6 $$\bar{{\rm{v}}}=\frac{{{\rm{D}}}^{2/3}}{{60}^{1/3}{{\rm{K}}}^{1/6}}$$

This is an important observation because it implies that Equation 5 defines motor primitives that scale with the movement size according to a two-third power law, i.e. $\bar{v}\propto {D}^{\alpha }$ with α = 2/3. Herein, we refer to α as the scaling exponent.

Experimental data consistent with Equation 6 would provide an interesting key to the interpretation of functional magnetic resonance data by Kadmon Harpaz *et al*.^31^ showing that human subjects’ brain activity is marked by a scale-invariant encoding of movement patterns. Results in line with this observation were published by Overduin *et al*. who showed first that invariant muscle synergies are associated with grasping objects of different size and/or shape^32^ and more recently that the generation of muscle synergies is associated with “cortical synergies” that are modulated consistently with the modulation of the muscle synergies^33^. Equation 6 suggests that the scale-invariant encoding of movement patterns observed by Kadmon Harpaz *et al*.^31^ in the brain activity and by Overduin *et al*.^32,33^ in the muscle synergies and the associated cortical synergies result in the scaling of motor primitives that mark the kinematics of motion. Accordingly, motor commands underlying the generation of upper-limb movements could be looked upon as consisting of two components: (1) a component aimed to generating scale-invariant motor primitives as shown in Equations 5 and (2) a component aimed to scaling the motor primitives according to Equation 6.

To explore the validity of the above-described theoretical model, we asked a group of ten healthy subjects to perform a battery of upper-limb motor tasks. We tracked their movements using a camera-based motion capture system (VICON, Oxford UK) and analyzed separately the x, y, and z-components of movement. For each component, we segmented the velocity time-series using the points of zero-crossing. We refer to the derived data segments as “movement elements”^26^, since the segmentation that we used is conceptually similar to the approach proposed by Brooks^27,28^ and later adopted by von Hofsten^25^, who referred to such data segments as “movement elements”. It is worth noticing that we used the same nomenclature used by von Hofsten^25^ even if he derived the movement elements by analyzing the magnitude of the velocity vector, whereas we analyzed individually the x, y, and z-components of movement. Then, we tested if the identified movement elements obeyed Equations 5 and 6. We estimated the Pearson correlation coefficient between the experimental data and the theoretical velocity profile for each movement element. We plotted the mean velocity value vs. the displacement for each movement element using a log-log scale. The slope of the regression line fitting the experimental points determined the scaling exponent for each motor task. Finally, we derived the value of the constant $K$ from Equation 4 for each movement element and computed its average value across the movement elements for each motor task.

Herein, we refer to the novel analysis method summarized above as the *movement element decomposition*. This technique was applied to data collected while subjects performed the following motor tasks: drawing ellipses, drawing complex two-dimensional shapes, handwriting, three-dimensional reaching movements, and three-dimensional random movements. The results showed that the motor patterns associated with these motor tasks can be looked upon as a combination of movement elements obeying Equations 5 and 6. This observation suggests that these tasks are performed as a combination of building blocks aimed to reach for specific target points in space. This was not totally unexpected for simple movements. However, it was surprising for complex movements, especially for three-dimensional random movements. To investigate if the proposed principle of movement generation is associated with the intention of reaching for target points in space, we performed two additional experiments. In the first experiment, we instructed subjects to reach for specific positions while sliding their hand along a ruler. In the second experiment, we instructed subjects to move their hand along the ruler until a visual cue indicated that they had to change the direction of movement. The results of these experiments showed that the intention of reaching for target points in space underlies the generation of movement elements.

This is the first study showing that a broad range of complex upper-limb movements are generated by combining motor primitives that scale with the size of movement according to a two-third power law (Equation 6). In the following, we provide a detailed report of the results of the study and discuss the implications of this newly discovered law underlying the generation of human movements of the upper limbs.

## Results

### Drawing Ellipses

Subjects drew five ellipses of different sizes by using printed templates positioned on a table. For each of the ellipses, subjects repeated five times the movement to trace each elliptical shape.

**Figure 1:**
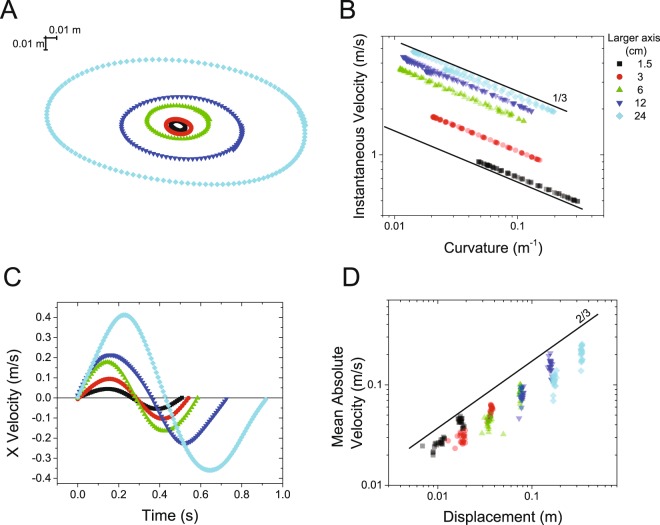
Analysis of the trajectories of motion performed by a subject while drawing ellipses of different sizes. Data for ellipses of different sizes is shown in different colors. (**A**) Trajectories of motion; (**B**) log-log plot of the instantaneous velocity vs. the curvature of the trajectory of motion: (**C**) x-component of the velocity of movement during one repetition of the drawing movement for each ellipse; and (**D**) log-log plot of the mean of the absolute value of the velocity for each movement element and the associated displacement (the line represents the power-law shown in Equation 6).

Figure 1A shows the elliptical movements tracked by a camera-based motion capture system using a reflective marker positioned on the pen used to trace the ellipses. Data is shown for one representative subject. Figure 1B highlights the relationship between the instantaneous velocity (i.e. the magnitude of the velocity vector) and the curvature of the movement for each of the ellipses. The log-log plot shows the one-third power law (Equation 1) relating the instantaneous velocity and the curvature of the movement for each of the ellipses.

Figure 1C shows the x-component of the velocity of movement during one repetition of the drawing movement for each of the five ellipses. Each repetition was marked by two movement elements as determined by the points of zero-crossing of the velocity time series. The movement elements have the shape predicted by Equation 5. Similar plots were derived for the y-axis. Figure 1D shows the log-log plot of the mean of the absolute value of the velocity for each movement element and the associated displacement values. Estimates for both the x and y components were used in this plot. The plot shows that the velocity of the movement elements scales with the magnitude of the displacement as predicted by the power law shown in Equation 6 with a scaling exponent close to the predicted value α = 2/3.

Figure 1 highlights that the one-third power law and the analysis via the *movement element decomposition* method capture two different aspects of the control of movement. The former captures the invariance to the size of the movement of the relationship between the instantaneous velocity and the movement curvature. The *movement element decomposition* captures the scaling of the mean of the absolute value of the velocity profile of the movement elements with the associated displacement.

Analysis of the group results (n = 10) showed that the correlation between the experimental data and the values of the velocity profiles predicted by Equation 5 was 0.77 ± 0.07 (mean ± standard deviation) across subjects. The slope of the regression line (i.e. the scaling exponent α) was 0.56 ± 0.12 with correlation equal to 0.89 ± 0.05. The value of $K$ was 0.43 ± 0.38.

### Drawing Complex Two-Dimensional Shapes

Subjects drew pure frequency curves. These curves obey the relationship *h(θ)* = *θ sin (νθ)* where *θ* is the angular direction of the tangent vector defined using a moving frame with unit tangent and normal vectors as the basis vectors (Frenet-Serret frame). ε is defined as the amplitude and *ν* as the frequency of the curve. Huh and Sejnowski^7^ used pure frequency curves to show that the one-third power law can be extended to complex geometrical shapes by defining a spectrum of power laws. We used pure frequency curves to test if the scaling phenomenon shown in Equation 6 held when subjects drew complex two-dimensional shapes.

**Figure 2:**
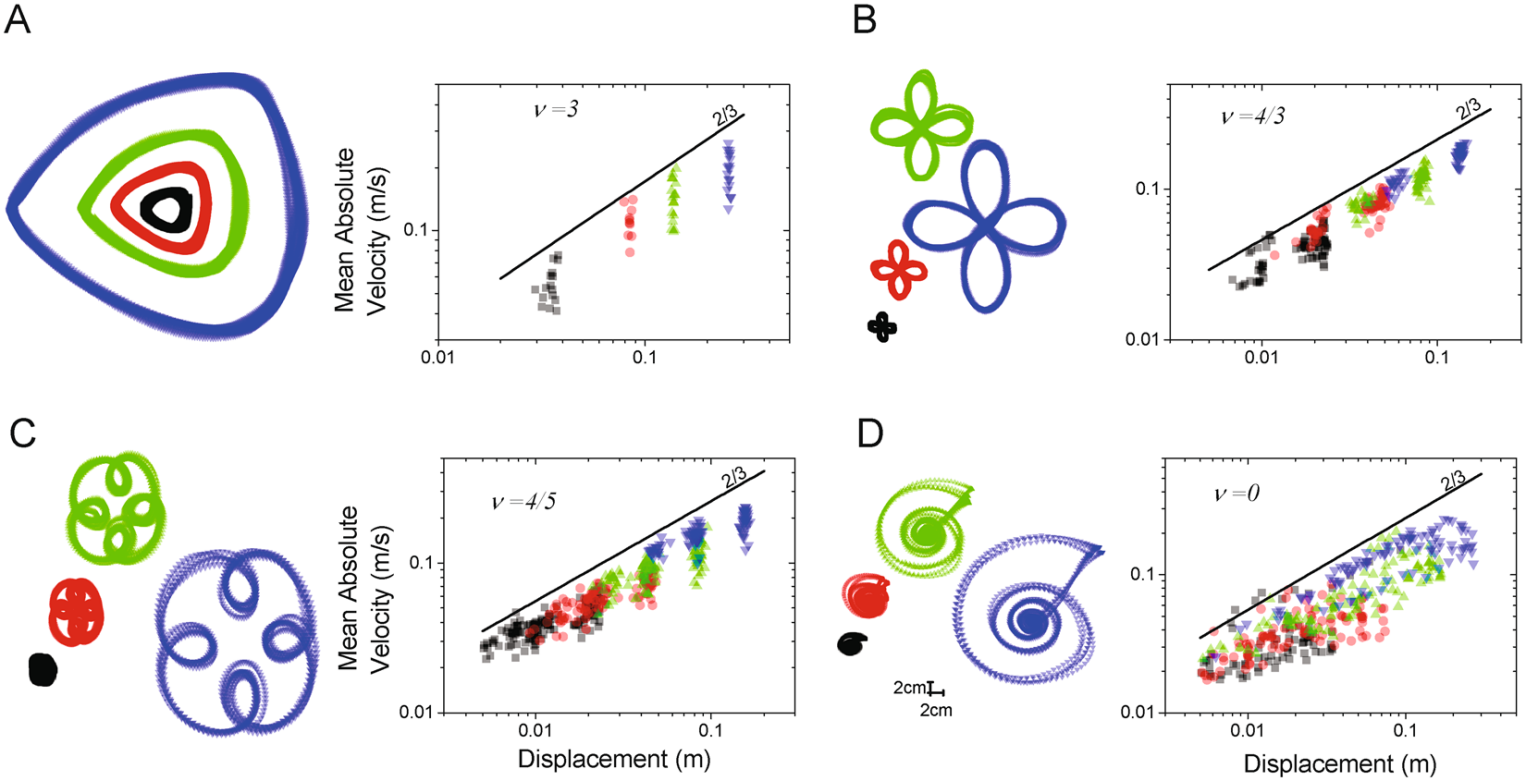
Analysis of pure frequency curves using the movement element decomposition method. Data is shown for ν = 3 (panel A), ν = 4/3 (panel B), ν = 4/5 (panel C), and ν = 0 (panel D). Each panel shows the traces detected by a camera-based motion capture system (left plot) and the log-log plot of the absolute value of the velocity of the movement elements vs. the corresponding displacement values (right plot) for one subject. The latter shows that the movement elements for all the pure frequency figures obey the power-law shown in Equation 6.

Figure 2 shows the results obtained from one representative subject. The movement elements for all subjects and all geometrical shapes were found to obey Equation 5. The correlation between the experimental data and the values derived from Equation 5 was 0.83 ± 0.04, 0.86 ± 0.04, 0.89 ± 0.05, and 0.86 ± 0.05, for ν = 3, ν = 4/3, ν = 4/5, and ν = 0, respectively.

The scaling phenomenon observed when subjects drew ellipses was observed for all the pure frequency curves. From the log-log plot of the mean of the absolute value of the velocity of the movement elements vs. the corresponding displacement values, we derived the slope of the regression lines (i.e. the scaling exponent α) for the group data and found it to be 0.54 ± 0.12, 0.69 ± 0.10, 0.63 ± 0.09, and 0.60 ± 0.11, for ν = 3, ν = 4/3, ν = 4/5, and ν = 0, respectively. The associated Pearson correlation coefficients were 0.79 ± 0.08, 0.91 ± 0.02, 0.87 ± 0.06, and 0.83 ± 0.08. The constant $K$ was found to be equal to 0.17 ± 0.30, 0.01 ± 0.01, 0.01 ± 0.01, and 0.04 ± 0.03.

### Handwriting

Subjects wrote the words “Boston” and “Harvard” in cursive and capital letters. The task was repeated three times. It is worth emphasizing that handwriting involves a combination of linear and curvilinear movements of various complexities.

**Figure 3:**
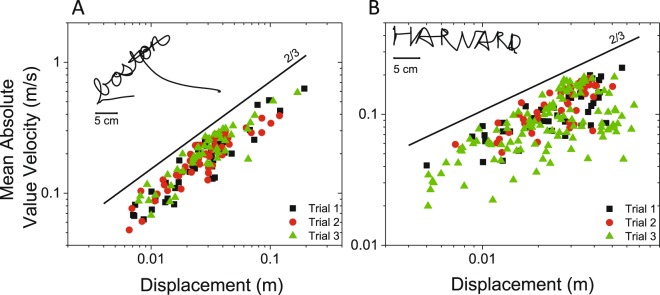
Analysis of handwriting data. The panels show the log-log plots of the absolute value of the velocity of the movement elements vs. the corresponding displacement values when one subject wrote the word Boston in cursive letters (left panel) and the word Harvard in capital letters (right panel). The plots show that the movement elements for the handwriting data obey the power-law shown in Equation 6.

Figure 3 shows an example of the results obtained from one representative subject. The plots show that the scaling phenomenon observed for drawing two-dimensional shapes applies also to handwriting. Analyses of the group data showed that the shape of the movement elements matched closely the values predicted by Equation 5. The correlation between observed and expected values was 0.88 ± 0.04, 0.84 ± 0.03, 0.86 ± 0.04, and 0.81 ± 0.06, when subjects wrote the words “Boston” and “Harvard” in cursive and capital letters, respectively. The slope of the regression lines derived from the log-log plots of the mean of the absolute value of the velocity of the movement elements vs. the corresponding displacement (i.e. the scaling exponent α) was 0.60 ± 0.04, 0.65 ± 0.08, 0.58 ± 0.09, and 0.62 ± 0.12, respectively. The associated correlation coefficients were 0.76 ± 0.08, 0.68 ± 0.11, 0.69 ± 0.11, and 0.64 ± 0.16. The constant $K$ was found to be equal to 1.54E-3 ± 2.42E-3, 1.92E-3 ± 2.69E-3, 2.95E-3 ± 5.87E-3, and 7.42 ± 0.01.

### Three-Dimensional Movements

We asked subjects to perform two sets of three-dimensional movements. The first set of movements consisted of reaching for and transporting a can of soda. Study participants were instructed to put their arm in a predefined starting position and to reach for and retrieve a can of soda placed on a table. They were asked then to move the can of soda to five different pre-set positions on the table and to place it each time back in the starting position. For the second set of movements, subjects were instructed to perform three-dimensional arm movements spanning randomly the space in front of them for a period of 15 s.

**Figure 4:**
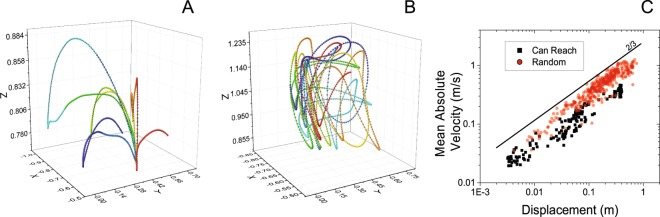
Analysis of three-dimensional movement data. Panel A shows an example of movement trajectories recorded during the performance of arm movements to reach and transport a can of soda (n = 1). Panel B shows an example of trajectories recorded during the performance of random movements. Panel C shows the absolute value of the velocity of the movement elements vs. the corresponding displacement values for the movements shown in the other two panels. The plot shows that the movement elements for all three-dimensional movement data obey the power-law shown in Equation 6.

Figure 4 shows an example of the results obtained from one representative subject. Figure 4A shows a sample of the three-dimensional trajectories of motion of the hand during the performance of the task consisting of reaching and transporting a can of soda. Figure 4B shows the hand trajectories for a sample of random movements. The movement elements derived for the x, y, and z movement components showed the same scaling phenomenon observed for two-dimensional movements (Fig. 4C).

Analysis of the group results showed that the movement elements matched well the values predicted by Equation 5. The correlation between observed and predicted values was 0.79 ± 0.05 for the task consisting of reaching and transporting a can of soda and 0.84 ± 0.03 for the random movements. The slope of the regression lines derived from the log-log plots of the mean of the absolute value of the velocity of the movement elements vs. the corresponding displacement (i.e. the scaling exponent α) was 0.64 ± 0.03 and 0.64 ± 0.05, respectively. The associated correlation coefficients were 0.94 ± 0.02 and 0.72 ± 0.05. The values of the constant $K$ were 3.10E-3 ± 2.32E-3 and 6.45E-4 ± 1.10E-3.

### One-Dimensional Movements with and without Targets

The observation that the movement elements derived from random three-dimensional movements obey the scaling phenomenon predicted by Equation 6 made us wonder if subjects moved in an apparent random manner but the underlying motor plan was equivalent to pointing at imaginary targets. To investigate this hypothesis, we asked subjects to move their hand along a ruler in the x-direction. For a set of trials (herein referred to as “movements with targets”), we asked subjects to move the hand from the current position to a new position in a sequence that we determined using a random number generator. In another set of trials (herein referred to as “movements without targets”), we asked subjects to move from the current position in one direction until instructed with a visual cue to change the direction of movement.

**Figure 5:**
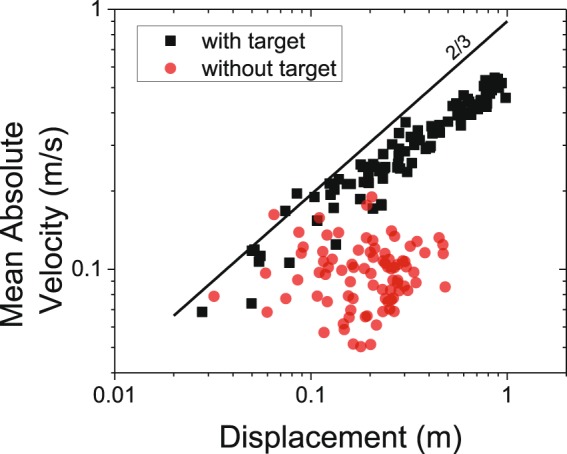
Analysis of one-dimensional movement data from a representative subject. The data for the movements with target obeys the power law shown in Equation 6, whereas the data for the movements without target does not obey such power law.

Figure 5 shows the results for one representative subject. The movement elements for the movements with targets show the scaling phenomenon. In contrast, the movement elements for the movements without targets show no association between the mean velocity and the corresponding displacement of the movement elements. Analysis of the group results showed that the movement elements matched well the velocity values predicted by Equation 5 for the movements with targets (correlation = 0.89 ± 0.03) but not for the movements without targets (correlation = 0.13 ± 0.13). The slope of the regression lines derived for different subjects from the log-log plots of the mean of the absolute value of the velocity of the movement elements vs. the corresponding displacement for the movements with targets (i.e. the scaling exponent α) was 0.55 ± 0.03 with correlation equal to 0.86 ± 0.07. In contrast, the data for the movements without targets showed no apparent correlation between the mean of the absolute value of the velocity of the movement elements and the corresponding displacement (correlation = 0.08 ± 0.08). The value of $K$ for the data derived from the movements with targets was 7.03E-3 ± 7.24E-3.

## Summary

**Figure 6:**
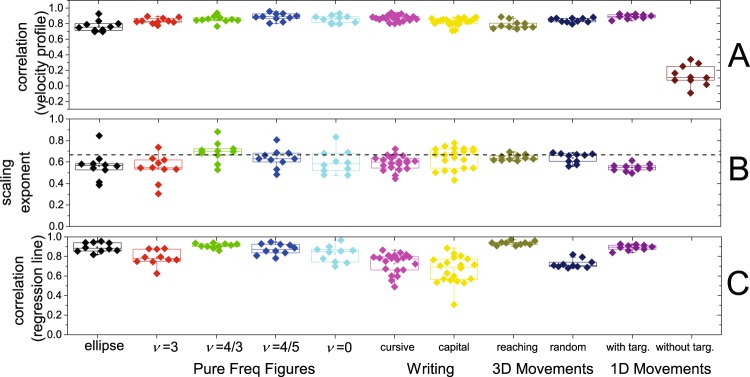
Summary of the results of the analyses performed for the data collected during the performance of all the motor tasks considered in the study (mean ± SD) for all ten subjects. Panel A - Correlation coefficients between the experimental data and the theoretical velocity profile (Equation 5) for each movement element. Panel B - slopes of the regression lines (i.e. scaling exponent) fitting the experimental points to determine the scaling properties of the movement elements (Equation 6). Panel C - correlation of the regression lines determining the scaling properties of the movement elements based on the experimental data.

Figure 6 and Table 1 provide a summary of the results of the study.

Figure 6A shows the values of the correlation coefficients estimated to assess if the movement elements matched the values predicted by Equation 5. The average correlation coefficient values ranged from 0.77 to 0.89, thus indicating that the movement elements fit well the velocity profiles predicted by Equation 5. The only task that did not appear to consist of movement elements obeying Equation 5 was the performance of one-dimensional movements without targets. A Friedman ANOVA test showed a significant difference (Friedman χ^2^(13) = 71.67, p ≅ 3.9 E-10) among the correlation coefficient values across tasks. Pairwise comparisons performed using the Conover post-hoc test for dependent samples showed consistent differences between the correlation coefficient values for the one-dimensional movements without targets vs. all other tasks. A few other differences were identified among the correlation coefficient values for the other tasks. However, the magnitude of such differences was small. Detailed results are shown in the Supplementary Information section (Table S1).

**Table 1:** Estimates of the average and standard deviation (SD) values of K derived from Equation 4 for the movement elements associated with the performance of all the motor tasks considered in the study for all subjects (n = 10).

	mean ± SD
Ellipse	4.43 ± 0.38
Pure Freq ν = 3	0.17 ± 0.30
Pure Freq ν = 4/3	0.01 ± 0.01
Pure Freq, ν = 4/5	0.01 ± 0.01
Pure Freq ν = 0	0.04 ± 0.03
Boston Cursive Letters	1.54E-3 ± 2.42E-3
Boston Capital Letters	1.92E-3 ± 2.69E-3
Harvard Cursive Letters	2.95E-3 ± 5.87E-3
Harvard Capital Letters	7.42E-3 ± 0.01
3D Can of Soda	3.10E-3 ± 2.32E-3
3D Random Movements	6.45E-4 ± 1.10E-3
1D with Targets	7.03E-3 ± 7.24E-3

Figure 6B shows that all the tasks that were successfully decomposed in movement elements obeying Equation 5 were marked by the above-discussed scaling phenomenon shown in Equation 6. The average scaling exponent value ranged from 0.54 to 0.69 across tasks. These results show a good approximation of the 2/3 value predicted for the scaling exponent α by Equation 6. The average Pearson correlation for the regression lines ranged from 0.64 to 0.94 across tasks (Fig. 6C). One-sample t-tests of the slope of the regression lines (i.e. the scaling exponent α) showed that the 95% confidence interval included the 2/3 value predicted by Equation 6 for most of the tasks. When the 95% confidence interval did not include the 2/3 value predicted by Equation 6, the value of the slope of the regression line was very close to 2/3. Detailed results are shown in the Supplementary Information section (Table S4).

Finally, Table 1 shows the average values of the constant $K$ derived from Equation 4. The values ranged from 6.45E-4 to 0.43 across tasks. It is worth noticing that the variability across subjects was very large, thus suggesting that subjects weigh differently the cost of time and the smoothness of movement (Equation 3). Detailed results are shown in the Supplementary Information section (Table S5).

## Discussion

The results of this study show that a broad range of upper-limb movements can be looked upon as a combination of movement elements obeying Equation 5 defined according to a Cartesian coordinate system with its axes oriented in the medio-lateral, antero-posterior, and vertical directions. Importantly, these movement elements scale with the magnitude of the associated displacement according to a newly discovered two-third power law (Equation 6). These findings add evidence to previous results suggesting that simple principles, such as the one-third power law^3,4^, govern the generation of human movements. Whereas the one-third power law captures the fact that the relationship between the instantaneous velocity of movement and its curvature is invariant to the size of the movement, the *movement element decomposition* approach - that we developed in this study - allowed us to unravel a new power law relating the mean of the absolute value of the velocity of the movement elements and the associated displacement. This power law underlies the generation of a broad range of linear and curvilinear movements and is the result of an optimization process that accounts for both the smoothness of movement^11^ and the cost of time^20–23^ as captured in Hoff’s work^24^ (Equation 3).

The results of our study are in line with previous work by von Hofsten^25^ showing that infants perform arm reaching movements by combining movement elements that become smoother and that are associated with a progressively larger displacement as infants improve their motor skills. Similar results were later published by Berthier^34^ and by Thelen *et al*.^35^. Our method differs from these studies in one important aspect, i.e. that it is based on the analysis of the components of movement along the axes of a Cartesian coordinate system oriented according to the anatomical planes. Previous studies relied instead on the analysis of the magnitude of the hand trajectory velocity vector. Our work shows that the combination of movement elements - that infants resort to as they learn how to perform arm reaching movements - marks the performance of a broad range of motor tasks in adults. Movement elements consistent with Equation 5 were observed for all the motor tasks tested in the study except for the one-dimensional movements without targets. The shape of the movement elements along the different axes was found to be very similar. Even when statistically significant differences were observed, such differences were modest in magnitude and the shapes of the movement elements for different axes closely resembled each other (see Figure S5 and Tables S2 and S3 in the Supplementary Information section).

The observation that complex upper-limb movements can be looked upon as a combination of movement elements marked by a velocity profile that is consistent with that associated with one-dimensional reaching movements^11^ has an interesting implication. It suggests that complex upper-limb movements can be thought of as a sequence of reaching movements with intermediate targets through which subjects reach the desired final position in space. Since we were able to identify the movement elements using a Cartesian coordinate system, one could argue that the results of this study are in support of a model of the motor control system relying on an internal representation of the surrounding space that is consistent with a Cartesian coordinate system with axes oriented in the antero-posterior, medio-lateral, and vertical directions. How the motor control system builds a model of the space surrounding the subject has been debated for a long time and recent studies have suggested that the motor control system might utilize different representations (e.g. coordinate systems) in different situations^36–38^. Our study does not exclude that other space representations might be suitable to identify movement elements that scale with the size of movement according to principles consistent with the power law that we have identified. However, using the above-defined Cartesian coordinate system allowed us to discover a simple power law that appears to underlie the generation of complex upper-limb movements. The scaling principle identified in the study is an attractive model to gain a better understanding of how the central nervous system may simplify the complex problem of controlling upper-limb movements.

It should be noted that the results reported in this manuscript are compatible with a variety of motor control schemas, ranging from models based on the interplay among simple movement parameters^39,40^ to complex models based on optimal feedback control theory^41,42^ and theories developed to explore the origin of intermediate movements^22,43,44^. However, the results of our study suggest that existing motor control schemas should be revisited by taking into account the fact that the motor output consists of a combination of movement elements that scale with the magnitude of the associated displacement. The results of our study may also shed new light on theories and models of motor learning. For instance, our results suggest that the prospective error model of motor learning based on motor primitives could be extended from simple reaching movements to complex multi-dimensional movements^45^.

Our results are also in line with experimental observations by Sosnik *et al*.^46^, who provided evidence of the existence of basic units of movement that subjects perform to completion even if told to stop right after the initiation of a movement unit. This observation appears to be consistent with the characteristics of the movement elements identified in this paper. Stopping the movement only after completion of the movement element that has been already initiated may be a strategy to preserve the smoothness of movement. Furthermore, this observation suggests that the movement elements discovered in our study are not simply an epiphenomenon of more complex, high-level control mechanisms associated with the generation of human movements, but they are basic building blocks utilized by the central nervous system to generate complex upper-limb movements.

The scaling of the movement elements with the size of movement provides further support to this statement. It is consistent with the invariance to the scale of movement observed in imaging data by Kadmon Harpaz *et al*.^31^. The authors showed that human subjects use a scale-invariant encoding of movement patterns. Specifically, they collected functional magnetic resonance data while subjects wrote letters of different size. They showed that accurate decoding of the letters was possible based on data patterns recorded from the primary motor cortex and the anterior intraparietal sulcus that were invariant to the size of the letters, hence suggesting that traditional hierarchical representations of the motor system may need to be revisited in favor of models based on more distributed networks of movement encoding^47^. Further evidence that the central nervous system generates movement patterns by scaling basic building blocks according to the size of movement was provided by Overduin *et al*.^32^. The authors showed that non-human primates grasp objects of different size and/or shape by relying on invariant muscle synergies and by modulating their recruitment strength and/or timing as needed to achieve the desired motor output. In a recent paper, Overduin *et al*.^33^ also showed that the generation of muscle synergies is associated with cortical activity that is organized according to “cortical synergies” that are modulated consistently with the muscle synergies.

The movement elements discovered in our study appear to be in line with these observations. We suggest that the invariance to the size of the movement performed by a subject of the brain activity as observed by Kadmon Harpaz *et al*.^31^ and of the scaling of muscle synergies as observed by Overduin *et al*.^32^. is related to the scaling of movement elements observed in our study. This would establish scaling of basic building blocks at the brain, muscle synergy, and kinematic levels as a fundamental mechanism utilized by the motor control system to simplify the process of generation of complex upper-limb movements. In the proposed schema, the generation of voluntary movements would not require a hierarchical model of the generation of upper-limb motor patterns in which abstract motor intentions are first encoded and then transformed into moment-by-moment encoding of movement kinematics. Rather, the motor areas encoding voluntary movements would be responsible for the generation of muscle synergies and associated kinematic motor primitives (i.e. movement elements) that obey Equation 5 and scale according to Equation 6, leading to the generation of complex movements. This implies that as a whole, the motor control system uses the strategy of scaling basic building blocks to minimize computational loads at different levels.

## Methods

### Participants

Ten healthy subjects (7 males; 26.4 ± 4.52 years of age; 9 right-handed) with no known neurological or orthopedic conditions affecting the control of motion were recruited to participate in the study. Written informed consent was obtained from all subjects. The protocol was approved by the Institutional Review Board of Spaulding Rehabilitation Hospital. The study was carried out in accordance with all relevant guidelines and regulations concerning the conduct of experiments involving human subjects.

### Experimental Procedures

Subjects were asked to perform a battery of motor tasks. They were instructed to sit at a table in a position such that the laboratory coordinate system was aligned with the anatomical planes (i.e. the x, y, and z axes of the laboratory coordinate system were oriented in the medio-lateral, antero-posterior, and vertical directions, respectively). For all the tasks, reflective markers enabling movement tracking were placed on the subjects’ sternum and the back of the dominant hand. For tasks when a pen was used, an additional reflective marker was placed on the tip of the pen. Tracking of the reflective markers was achieved by using a camera-based motion capture system (VICON, Oxford UK). The motor tasks included drawing different geometrical shapes, writing words in cursive and capital letters, performing three-dimensional arm reaching movements, performing three-dimensional random arm movements, and performing one-dimensional arm movements (i.e. sliding the hand along a ruler) with and without targets.

#### Drawing ellipses

Subjects were asked to trace a set of ellipses printed on an A3 size piece of paper positioned on the table. They were instructed to use a pen to trace the ellipses and to move the arm at comfortable speed. The ellipses were of 5 different sizes with the major axis of length 1.5, 3, 6, 12, and 24 cm, respectively. The tracing movement for each ellipse was repeated 5 times in a continuous fashion.

#### Drawing pure frequency figures

To test subjects while performing complex two-dimensional movements, we used pure frequency figures. Subjects were asked to use a pen to trace the figures and to move their arm at comfortable speed. Figures of different sizes were printed on A3 size pieces of paper and positioned on the table in front of the subjects to provide a template for the tracing movements. The figures were printed in four different sizes with width equal to 1.5, 3, 6, and 12 cm, respectively. The tracing movement for each figure was repeated 5 times in a continuous fashion for each size of the figure.

#### Hand-writing

Subjects were asked to write on a piece of paper the words “Boston” and “Harvard” in lower-case cursive and upper-case capital letters. Each word was written 3 times in cursive and capital letters.

#### Three-dimensional arm reaching movements

A can of soda was placed on the table in a set position to the right of the subject within arm-reaching distance. Subjects were asked to place their hand on the table in a marked position (herein referred to as “starting position”) right in front of them and close to the edge of the table. They were then asked to reach for the can of soda and move it to the position marked on the table as the starting position. Then, they were asked to move the can of soda between the starting position and five positions marked on the table. The task was repeated three times.

#### Three-dimensional random arm movements

Subjects were asked to perform three-dimensional random movements while holding a pen (as if scribbling in the air) for an interval of 15 s. The movement task was repeated three times.

#### One-dimensional movements with and without targets

A ruler with numbers from 0 to 60 spaced 2.5 cm apart was placed on the table. For the movements with targets, a set of 45 randomly assigned numbers between 1 and 60 were shown sequentially to the subjects using a computer screen. Subjects were asked to move a pen to the respective value on the ruler following the edge of the ruler. This task was repeated two times using different random sequences. For the movements without targets, subjects were asked to continuously move the pen along the edge of the ruler in the direction of an arrow shown on a computer screen. Subjects were asked to change the direction of movement when the direction of the arrow changed on the screen. Forty-five changes in the direction of the arrow were programmed. The sequence was programmed to ensure variable movement times. This task was repeated two times. Note that the first sequence and the second sequence of movement times were different.

### Data Processing

Data collected using the camera-based motion capture system (VICON, Oxford UK) was preprocessed using the system’s software to reconstruct the three-dimensional position of the reflective markers. Then, we transferred the data in Matlab (The MathWorks Inc, Massachusetts USA). First, we applied a low-pass filter with cut-off frequency equal to 10 Hz to attenuate noise components affecting the x, y, and z components of the reflective marker trajectories. Then, we derived the velocity profiles for each of the three axes and determined the time support of each movement component by detecting the zero-crossing points of the velocity time series. Finally, as previously proposed by Teulings *et al*.^48^ and by Rand *et al*.^49^, we applied task-specific criteria to discard movement elements that could not be detected reliably given the signal-to-noise ratio of the reflective marker trajectory time series. In our work, these criteria were based on the minimum displacement, duration, and velocity that could be detected on a task-by-task basis given the magnitude of the noise associated with each recording. The minimum displacement threshold was set to 5 mm for the figure-drawing and handwriting tasks and to 3 mm for the three-dimensional movements. For the one-dimensional movements, the minimum displacement threshold was set to 2 cm because it is known that one-dimensional point-to-point movements are marked by a single movement element and because the minimum displacement associated with sliding the hand along the ruler corresponded to adjacent marks on the ruler, which were spaced 2.5 cm apart. The minimum duration threshold was set to 100 ms for the figure drawing, the handwriting, and the three-dimensional movements and it was set to 200 ms for the one-dimensional movements. The minimum velocity threshold was set to 1 cm/s for all the tasks. It is worth emphasizing that we assessed the robustness of the data processing procedures and found them to be robust to additive noise. Detailed results are shown in the Supplementary Information section (Figures S1, S2, and S3).

### Statistical Analyses

Statistical analyses were performed using the R software package^50^. Descriptive statistics such as mean and standard deviation were computed for all the data. Results are reported throughout the manuscript in the format *mean* ± *standard deviation*. We tested if the shape of the observed movement elements for each task was different from the theoretical shape (i.e. Equation 5) (Fig. 6A) using the Friedman test. Post hoc analyses for relevant comparisons were performed using the Conover test^51^. Non-parametric tests were utilized due to the distribution of the data. To evaluate if the mean slope of the log-log plot of the mean of the absolute value of the velocity of the movement elements vs. the corresponding displacement (i.e. the scaling exponent α) was significantly different from the theoretical two-third value predicted by Equation 6 (Fig. 6B), we performed a two-tailed one-sample t-test for each task. Normality was assessed for all comparisons. We used a one-sample t-test to compare the theoretical and experimental data and did not adjust for multiple comparisons because we performed these comparisons independently even though they are presented in the same graph (Fig. 6B). The significance level was set a priori to p < 0.05.

### Data and code availability

The data collected in this study is available in PhysioNet https://physionet.org/. The code used in the study is available upon request to the corresponding author.

## Electronic supplementary material

Supplementary Information
