# Supplementary material for: Complex Upper-Limb Movements Are Generated by Combining Motor Primitives that Scale with the Movement Size
Source: Sci Rep. 2018 Aug 27;8:12918. doi: 10.1038/s41598-018-29470-y (PMC6110807; doi:10.1038/s41598-018-29470-y)
Supplement: Supplementary file 1 — Supplementary Information [file 41598_2018_29470_MOESM1_ESM.pdf]

Supplementary Information

Complex Upper-Limb Movements Are Generated by Combining Motor Primitives that Scale with the Movement Size

Jose Garcia Vivas Miranda[1,2 *], Jean-François Daneault[2], Gloria Vergara-Diaz[2,], Ângelo Frederico Souza de Oliveira e Torres[1], Ana Paula Quixadá[1], Marcus de Lemos Fonseca[3], João Paulo Bomfim Cruz Vieira[1], Vitor Sotero dos Santos[1], Thiago da Cruz Figueiredo[1,4], Elen Beatriz Pinto[5], Norberto Peña[1], Paolo Bonato[2,6]

[1] Institute of Physics, Laboratory of Biosystems, Universidade Federal da Bahia, Salvador BA, Brazil

[2] Department of Physical Medicine and Rehabilitation, Harvard Medical School, Spaulding Rehabilitation Hospital, Boston MA, USA

[3] Faculdade Social da Bahia, Salvador BA, Brazil

[4] Institute of Medical Psychology and Behavioural Neurobiology, University of Tübingen, Tübingen, Germany

[5] Motor Behavior and Neurorehabilitation Research Group, Bahiana School of Medicine and Public Health, Salvador BA, Brazil

[6] Wyss Institute for Biologically Inspired Engineering, Harvard University, Boston MA, USA

Robustness to Additive Noise of the Movement Element Decomposition Method

To assess the robustness to additive noise of the movement element decomposition method, we generated simulated time series with elements marked by a velocity profile $\mathrm{v}(.)$ determined according to *Equation 5* of the main manuscript, which is reported below:

$$\mathrm{v(t)} = \mathrm{D}\left[\frac{30}{\mathrm{t_f}^5}\,\mathrm{t}^4 - \frac{60}{\mathrm{t_f}^4}\,\mathrm{t}^3 + \frac{30}{\mathrm{t_f}^3}\,\mathrm{t}^2\right] \quad \text{(S1)}$$

In this equation, t represents time, t_f the duration of the movement element, and D the displacement associated with the movement element. We simulated three sets of 100 time-series corresponding to unidimensional movement trajectories consisting of 30 movement elements obeying *Equation S1*. The velocity profiles were scaled using a "normalized" scaling exponent (i.e. α=1). The value of the displacement D was generated randomly with uniform distribution between a minimum value D_{min} and a maximum value D_{max}. The minimum displacement was the same for all the sets of simulated time-series ($D_{min} = 5\ mm$). The maximum displacement was different for each set as follows: $D_{max} = 2\ m$ for the set of simulations whose results are shown in *Figure S1*, $D_{max} = 1\ m$ for those shown in *Figure S2*, and $D_{max} = 0.2\ m$ for those shown in *Figure S3*. The simulated sampling rate was equal to 120 Hz, namely the same as the sampling rate of the actual recordings obtained using the camera-based motion capture system utilized in the study. We randomly positioned the movement elements in time, with the constraint that movement elements would not overlap in time. We then added uniformly distributed random noise of amplitude ranging from 1E-4 to 0.5 m to assess the robustness of the movement element decomposition method to additive noise.

Noise levels up to 1 mm in amplitude did not significantly affect the estimation of the relationship between the mean of the absolute value of the velocity of the movement elements and the corresponding displacement (panel A of each figure), the number of movement elements identified by the algorithm (panel B), and the peaks detected for each movement element (panel C). These results show that the proposed technique is suitable to process recordings carried out using a camera-based motion capture system, which are typically affected by noise of amplitude smaller than 1 mm.

Movement Elements Are Defined According to the Anatomical Planes

Previous work showed that simple one-dimensional point-to-point movements are marked by a velocity profile with shape that obeys *Equation S1*. However, previous investigators failed to identify movement elements with such velocity profile when they analyzed complex upper-limb movements. We argue that this is the case because previous studies attempted to identify movement elements by analyzing the magnitude of the velocity vector associated with the trajectory of movement. *Figure S4* shows examples of the velocity profiles that we estimated from data collected while subjects performed two of

the tasks chosen for the study, i.e. drawing an ellipse (panel A) and drawing a spiral (panel B). The figure shows both the magnitude of the velocity vector and the velocity along the x- and y-components of the movement trajectory, where x and y are the axes of a Cartesian coordinate system oriented according to the anatomical planes in the medio-lateral and antero-posterior directions, respectively.

It is worth emphasizing that movement elements of shape consistent with *Equation S1* are only apparent when one observes the velocity trajectories along the x- and y-components of movement. In contrast, the magnitude of the velocity vector displays multiple peaks that are difficult to relate to the peaks of the movement elements along the x and y axes. A simple relationship between the magnitude of the velocity vector and the velocity trajectory along the x- and y-components of movement would be apparent only if the movement elements along the x and y axes happened to be synchronous, i.e. if they occurred at the same time. However, this can only be possible for linear movements, but not for curvilinear movements. Besides, estimating the magnitude of the velocity vector requires combining the velocity of the x- and y-components of the movement trajectory via a quadratic transformation. This makes it challenging to decompose the movement velocity trajectory in its components without constraining the algorithm to search for solutions marked by two distinct components along the x and y axes of movement.

The movement elements that we discovered by analyzing the x- and y-components of the movement trajectory were found to be consistent across all the tasks that we tested in the study, irrespective of the fact that the task consisted of a linear or a curvilinear movement. The only exception to this rule was the data collected during the performance of the one-dimensional movements without targets.

Table S1 shows the differences observed across tasks in how closely the movement elements identified for each task matched the theoretical velocity profile shown above (*Equation S1*). Specifically, we compared the correlation coefficients between the theoretical velocity profile and the experimental data using the Friedman ANOVA test as reported in the main manuscript. Furthermore, we performed Conover post-hoc tests for dependent samples to compare the correlation coefficients across tasks. The post-hoc tests showed consistent differences between the correlation coefficient values for the one-dimensional movements without targets vs. all other tasks. A few other differences were identified among the correlation coefficient values for the other tasks. However, the magnitude of such differences was small as shown in Figure 6A of the main manuscript where average correlation coefficients ranging from 0.77 to 0.89 are shown, thus indicating that the movement elements match well the velocity profiles predicted by *Equation S1*.

Interestingly, not only the movement elements were shown to be very similar across tasks, but also they were shown to be very similar across axes. In other words, the shape of the movement elements was the same for the x- and y-components of the movement trajectory for planar movements and for all three

axes of movement (i.e. the x-, y-, and z-components of movement) for three-dimensional movements. Results of statistical tests performed for planar movements are shown in *Table S2*, which summarizes the results of the Wilcoxon tests performed to compare the velocity profiles of the x and y components of movement. Similarly, *Table S3* shows the results of the analysis of three-dimensional movements obtained by performing Friedman ANOVA tests performed to compare the velocity profiles of the movement elements identified by analyzing separately the x-, y-, and z-components of movement. In the few cases in which a statistically significant difference was identified, the magnitude of such difference was very modest.

<u>An Optimization Process Underlies the Generation of Upper-Limb Movements</u>

Our experimental observations unraveled an optimization process underlying the generation of upper-limb movements. In fact, not only the movement elements that we derived using the proposed method displayed the predicted shape shown in *Equation S1*, but the identified movement elements also scaled with the size of movement according to Equation 6 of the main manuscript, which is reported below:

$$\bar{v} = \frac{D^{2/3}}{60^{1/3}K^{1/6}} \tag{S2}$$

In this equation, $\bar{v}$ is the mean velocity of movement, D is the displacement associated with each movement element, and K is a constant whose value determines the relevance of the smoothness of movement compared to the cost of time as captured by the duration of the movement element t_f as shown below:

$$I = t_f + K\int_{t=0}^{t=t_f}(u_x^2 + u_y^2)dt \tag{S3}$$

In other words, our experimental observations indicated that human subjects generate upper-limb movements according to the cost function shown above (*Equation S3*) thus accounting for both the smoothness of movement and the cost of time. Optimization of this cost function leads to the generation of upper-limb movements via the combination of movement elements with shape determined by *Equation S1* that scale with the size of the elements (i.e. the distance D spanned by each movement element) according to *Equation S2*. This equation shows a power relationship between average velocity $\bar{v}$ and displacement D of the movement elements, i.e. $\bar{v} \propto D^{\alpha}$ with α=2/3. Herein, we refer to α as the scaling exponent.

Table S4 shows the results of statistical tests demonstrating that, when motor tasks consisted of movement elements obeying *Equation S1*, the mean of the absolute value of the velocity of the movement elements and the corresponding displacement obeyed a power law as predicted by *Equation S2*. In fact, one-sample t-tests allowed us to determine that the 95% confidence interval of the slope of the regression

lines (i.e. the value of the scaling exponent α) derived from the log-log plots of the mean of the absolute value of the velocity vs. the associated displacement of the movement elements included the 2/3 value predicted according to *Equation S2* for virtually all the tasks. Even when this was not the case, the 95% confidence interval was very close to include the 2/3 value predicted according to *Equation S2*.

Interestingly, whereas the optimization principle utilized by all subjects to generate the movement elements appears to be consistent across subjects and results in a smooth movement output, the approach to the trade-off between the cost of time and the smoothness of movement (i.e. *Equation S3*) seems to vary significantly across subjects. *Table S5* shows the average K values estimated from *Equation S2* for each subject and each motor task. The estimated average K values vary significantly across subjects. Interestingly, the table suggests that different optimization trade-off approaches are adopted by different subjects. In other words, while some subjects appear to prefer achieving the tasks in a short time period (i.e. they prioritize minimizing the cost of time), others appear to prefer achieving a smooth movement and hence they weigh less the cost of time.

<u>*Movement Elements as Motor Primitives*</u>

We look upon the movement elements discovered in this study as motor primitives underlying the generation of a broad range of upper-limb motor tasks. Interestingly, the characteristics of the identified movement elements suggest that complex upper-limb movements consist of elements aimed to reach for intermediate target points along the axes of the trajectory of movement. This observation is consistent with the way infants learn how to perform arm reaching movements. It is also consistent with recent literature that has shown that brain activity associated with a motor task such as writing is invariant to the scale of movement. In this context, the movement elements discovered in the study might be considered the kinematic basis of the scale-invariant encoding of movement patterns.

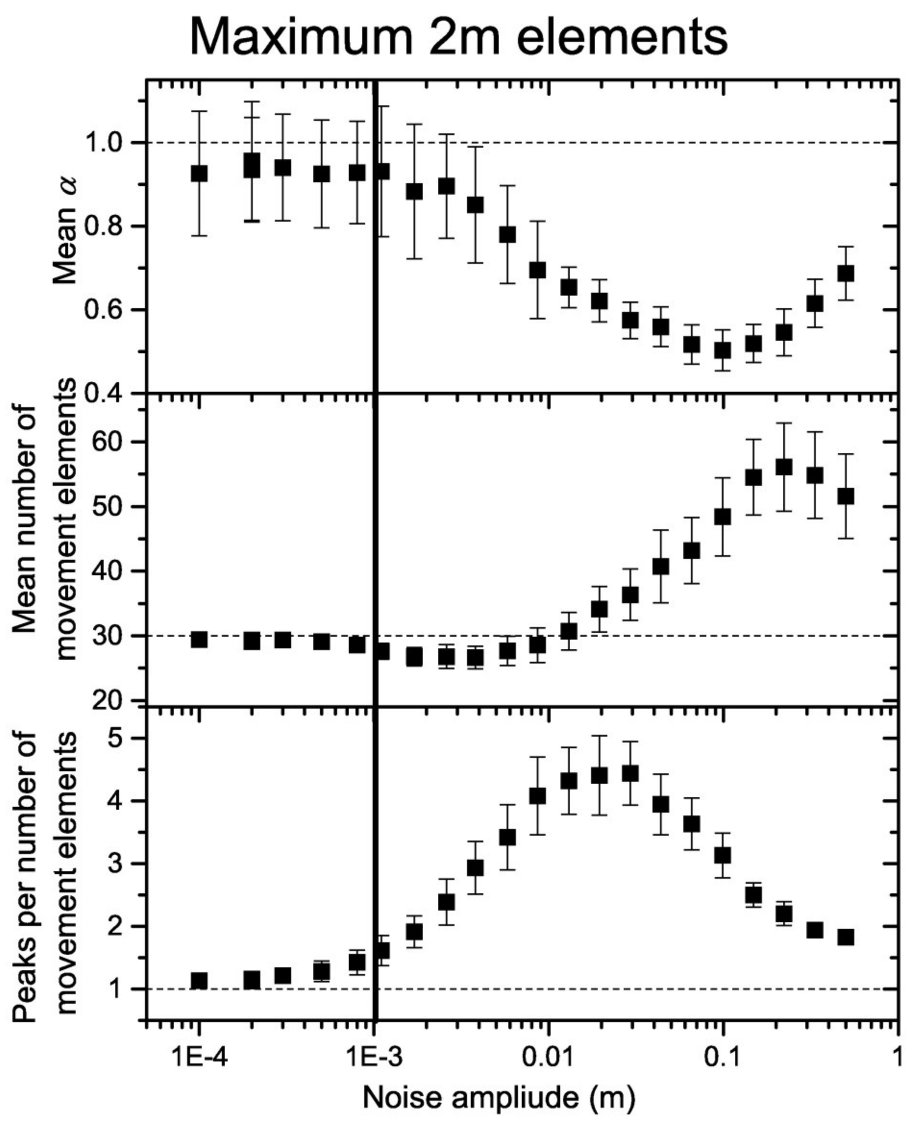

Figure S1. *Results of the simulations performed to assess the sensitivity of the movement element decomposition algorithm to additive noise for simulated movement elements ranging from a minimum of 5 mm to a maximum of 2 m. Results are shown as average and standard deviation values. Panel A – mean slope of the log-log plot of the mean of the absolute value of the velocity of the movement elements vs. the corresponding displacement. We used a "normalized" simulated scaling exponent (i.e.* $\alpha=1$*). Panel B – number of detected movement elements. We synthesized 30 movement elements according to Equation S1 for each simulated time-series. Panel C – average number of peaks detected for each movement element. Each synthesized movement element had a single peak. Spurious peaks were detected as the amplitude of the additive noise component increased.*

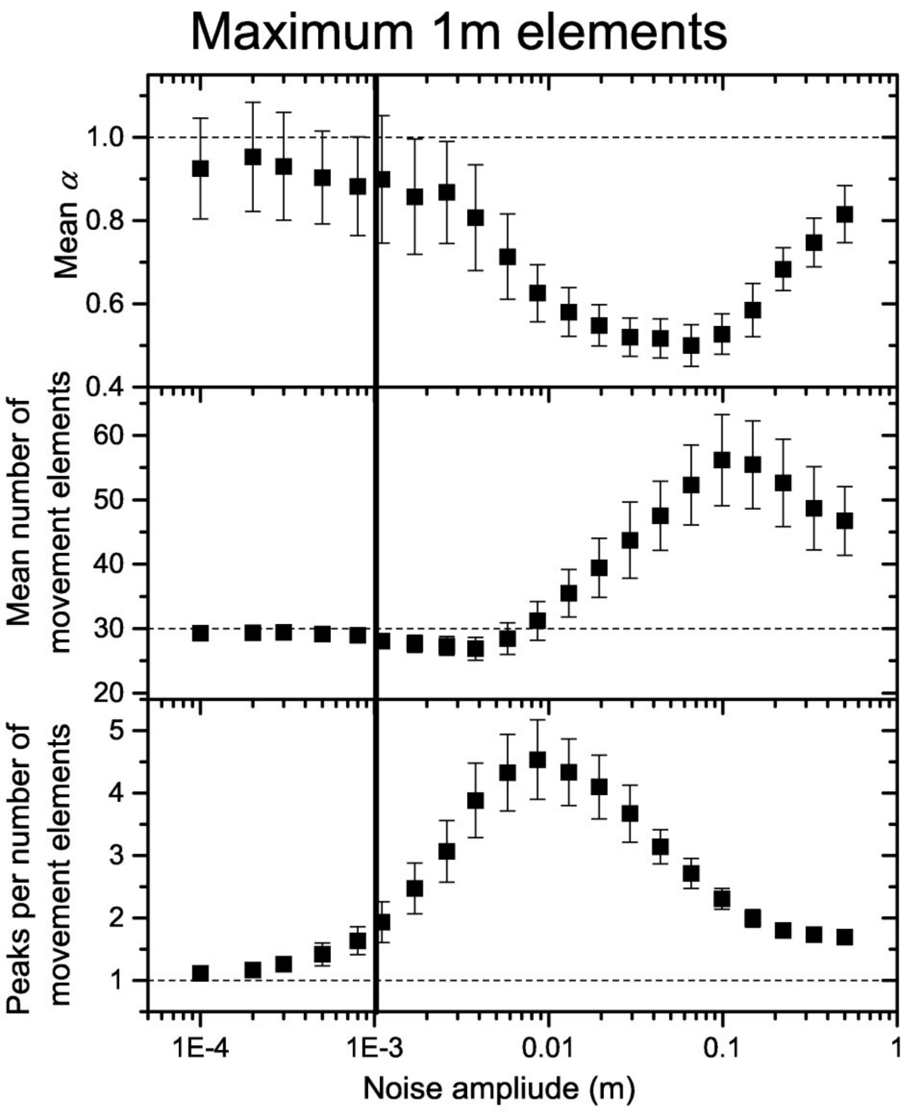

Figure S2. *Results of the simulations performed to assess the sensitivity of the movement element decomposition algorithm to additive noise for simulated movement elements ranging from a minimum of 5 mm to a maximum of 1 m. Results are shown as average and standard deviation values. Panel A – mean slope of the log-log plot of the mean of the absolute value of the velocity of the movement elements vs. the corresponding displacement. We used a "normalized" simulated scaling exponent (i.e. $\alpha=1$). Panel B – number of detected movement elements. We synthesized 30 movement elements according to Equation S1 for each simulated time-series. Panel C – average number of peaks detected for each movement element. Each synthesized movement element had a single peak. Spurious peaks were detected as the amplitude of the additive noise component increased.*

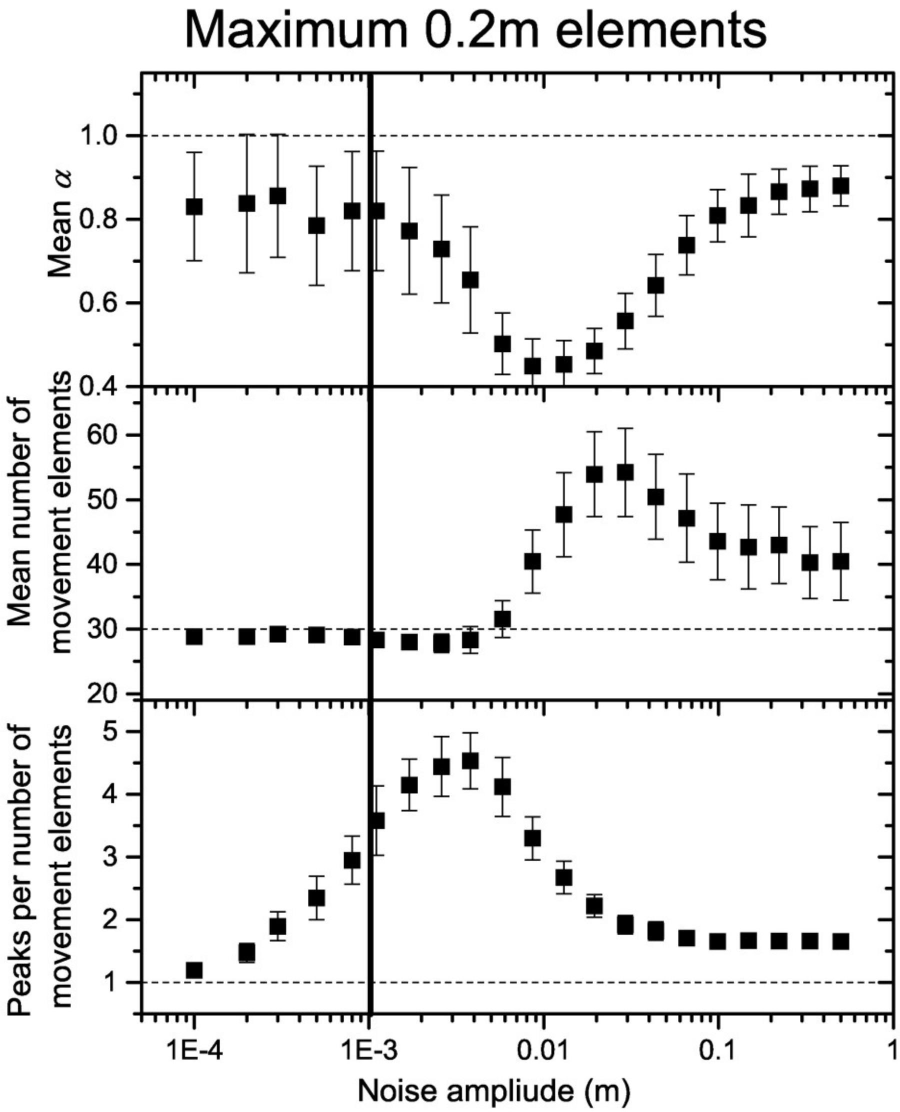

Figure S3. *Results of the simulations performed to assess the sensitivity of the movement element decomposition algorithm to additive noise for simulated movement elements ranging from a minimum of 5 mm to a maximum of 0.2 m. Results are shown as average and standard deviation values. Panel A – mean slope of the log-log plot of the mean of the absolute value of the velocity of the movement elements vs. the corresponding displacement. We used a "normalized" simulated scaling exponent (i.e.* $\alpha=1$*). Panel B – number of detected movement elements. We synthesized 30 movement elements according to Equation S1 for each simulated time-series. Panel C – average number of peaks detected for each movement element. Each synthesized movement element had a single peak. Spurious peaks were detected as the amplitude of the additive noise component increased.*

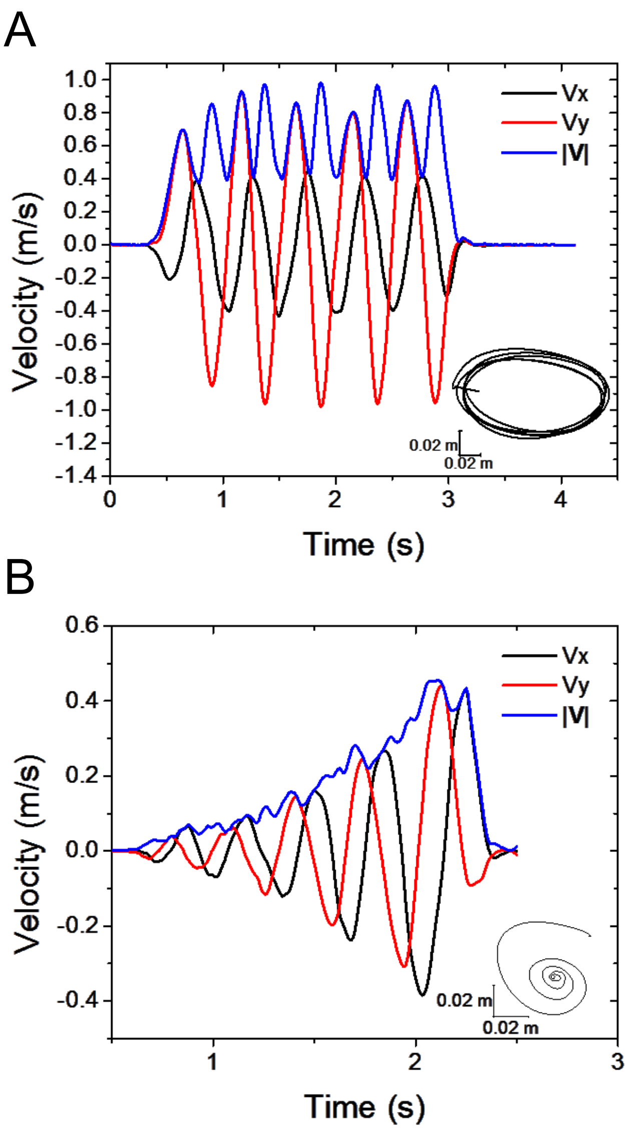

Figure S4. *Comparison of the velocity profiles derived from data collected while a subject drew an ellipse (panel A) and a spiral (panel B) using the magnitude of the velocity vector and the x- and y-components of the movement trajectory.*

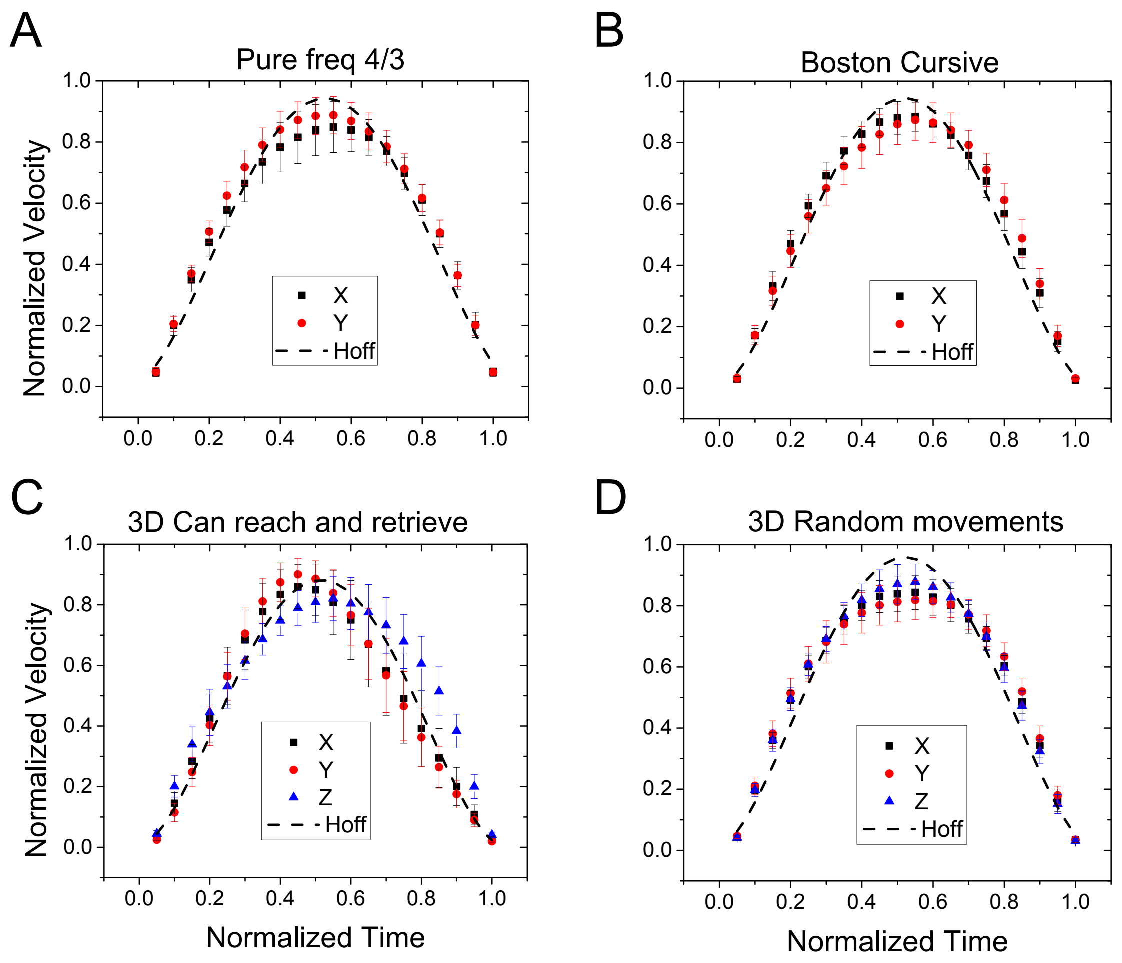

Figure S5. *Velocity profiles of the movement elements for different motor tasks. The data is shown for drawing a pure frequency curve for ν=4/3 (panel A), handwriting the word Boston in cursive letters (panel B), reaching for and transporting a can of soda (panel C), and performing random three-dimensional movements (panel D). All the movement elements for these tasks obey Equation S1 and are of nearly the same shape across axes.*

	Ellipse	Pure Freq ν=3	Pure Freq ν=4/3	Pure Freq , ν=4/5	Pure Freq ν=0	Boston Cursive Letters	Boston Capital Letters	Harvard Cursive Letters	Harvard Capital Letters	3D Can of Soda	3D Random Movements	1D with Targets	1D without Targets
Ellipse		0.84	4.9E-4	0.07	0.65	0.74	0.03	0.79	0.22	0.06	0.08	0.40	1.8E-3
Pure Freq ν=3			2.4E-4	0.04	0.51	0.60	0.02	0.65	0.15	0.04	0.05	0.51	3.3E-3
Pure Freq ν=4/3				0.08	2.2E-3	1.4E-3	0.17	1.2E-3	0.02	0.09	0.07	2.1E-5	5E-10
Pure Freq , ν=4/5					0.17	0.14	0.70	0.12	0.56	0.95	0.95	8.5E-3	1.8E-6
Pure Freq ν=0						0.90	0.08	0.84	0.43	0.15	0.19	0.19	3.9E-4
Boston Cursive Letters							0.06	0.95	0.36	0.12	0.15	0.24	6.1E-4
Boston Capital Letters								0.05	0.33	0.74	0.65	2.7E-3	3.3E-7
Harvard Cursive Letters									0.33	0.10	0.14	0.27	7.6E-4
Harvard Capital Letters										0.51	0.60	0.04	2.1E-5
3D Can of Soda											0.90	7.1E-3	1.4E-6
3D Random Movements												0.01	2.4E-6
1D with Targets													0.02
1D without Targets													

Table S1. *Results of Conover post-hoc tests comparing the correlation coefficients estimated for different tasks (Friedman chi-squared=71.67, df=13, p=3.95E-10). The correlation coefficients were computed to assess if the movement elements for each task obeyed Equation S1. The cells highlighted in yellow are those for which the test identified a significant difference.*

	W and *Z*	Median value of the difference	p-value x-axis vs. y-axis
Ellipse	W=10, Z=-3.53	11 %	3.9 E-4 **
Pure Freq $\nu=3$	W=44, Z= -2.26	NA	2.3 E-2
Pure Freq $\nu=4/3$	W=4, Z= -3.75	4 %	1.6 E-4 **
Pure Freq , $\nu=4/5$	W=46, Z= -2.15	NA	3.0 E-2
Pure Freq $\nu=0$	W=76, Z= -1.06	NA	0.28
Boston Cursive Letters	W=111, Z= 0.21	NA	6.7 E-2
Boston Capital Letters	W=56, Z= -1.81	NA	0.82
Harvard Cursive Letters	W=135, Z= 1.10	NA	0.60
Harvard Capital Letters	W=119, Z= 0.50	NA	0.26

Table S2. *Results of the Wilcoxon tests (W - sum of the signed ranks; Z - z-score for the test) for dependent samples performed to compare the velocity profiles of the x- and y-axis movement elements identified for the two-dimensional movements tested in the study. ** indicates when the obtained p-value was considered significant. Please notice that a Bonferroni adjustment was performed. When a statistically significant difference was detected, the table provides the median value of the percentage difference between the velocity profiles of the x- and y-axis movement elements. NA = not applicable.*

	Friedman X^2	p-value
3D Can of Soda	3.9	0.14
3D Random Movements	2.8	0.25

Table S3. *Results of the Friedman tests performed to compare the velocity profiles of the x-axis, y-axis, and z-axis movement elements identified for the three-dimensional movements tested in the study.*

	t(df)	p-value true mean ≠ 0.66	95% confidence interval
Ellipse	t(9)= -2.26	0.05	0.49 – 0.66
Pure Freq ν=3	t(9)= -2.61	0.03	0.49 – 0.65
Pure Freq ν=4/3	t(9)= 1.24	0.25	0.63 – 0.77
Pure Freq , ν=4/5	t(9)= -0.89	0.39	0.57 – 0.70
Pure Freq ν=0	t(9)= -1.70	0.12	0.52 – 0.68
Boston Cursive Letters	t(9)= -5.24	5.4E-4	0.57 – 0.62
Boston Capital Letters	t(9)=-0.24	0.82	0.60 – 0.71
Harvard Cursive Letters	t(9)= -2.71	0.02	0.52 – 0.65
Harvard Capital Letters	t(9)= -1.03	0.33	0.54 – 0.71
3D Can of Soda	t(9)= -2.06	0.07	0.62 – 0.66
3D Random Movements	t(9)= -1.72	0.12	0.59 – 0.67
1D with Targets	t(9)= -10.58	2.2E-6	0.52 – 0.57

Table S4. *Results of one-sample t-tests to determine if the 95% confidence interval of the slope of the regression lines derived from the log-log plots of the mean of the absolute value of the velocity vs. the associated displacement of the movement elements included the 2/3 value predicted according to Equation S2. It is worth emphasizing that the regression line was not derived for the data recorded during the performance of 1D movements without target because the data was not marked by movement elements consistent with Equation S1.*

	Ellipse	Pure Freq v=3	Pure Freq v=4/3	Pure Freq , v=4/5	Pure Freq v=0	Boston Cursive Letters	Boston Capital Letters	Harvard Cursive Letters	Harvard Capital Letters	3D Reach and retrieve	3D Random Movements	1D with Targets
Subject #1	0.11	0.03	0.01	8.32E-3	0.07	9.81E-4	2.55E-3	2.48E-3	5.67E-3	2.98E-3	2.17E-4	1.75E-3
Subject #2	0.60	0.97	0.04	0.04	0.07	3.44E-3	9.12E-3	3.38E-3	4.46E-2	6.54E-3	3.72E-3	7.52E-3
Subject #3	1.21	0.06	8.33E-3	3.38E-3	0.03	7.77E-3	1.60E-3	1.93E-2	1.43E-2	8.21E-4	6.54E-4	1.86E-3
Subject #4	1.46E-4	5.90E-5	6.80E-5	2.90E-5	7.50E-5	1.36E-4	8.70E-5	7.40E-5	2.96E-4	2.49E-3	1.60E-5	1.14E-3
Subject #5	0.08	0.05	0.02	9.66E-3	0.03	1.85E-4	2.35E-3	4.72E-4	5.96E-3	4.92E-3	2.15E-4	1.24E-2
Subject #6	0.63	0.36	1.41E-3	3.81E-3	0.03	3.07E-4	3.52E-4	2.19E-4	7.66E-4	9.30E-5	3.30E-5	2.49E-2
Subject #7	0.61	0.15	0.02	0.01	0.05	3.34E-4	2.57E-4	6.29E-4	7.91E-4	9.51E-4	2.01E-4	5.65E-3
Subject #8	0.70	0.04	0.01	5.30E-3	0.09	1.78E-3	1.20E-3	2.29E-3	8.69E-4	1.86E-3	4.55E-4	2.37E-3
Subject #9	0.17	0.03	5.83E-3	2.45E-3	5.86E-3	1.93E-4	1.60E-3	5.55E-4	7.61E-4	6.61E-3	6.46E-4	8.24E-3
Subject #10	0.22	4.08E-3	2.91E-3	3.88E-4	2.19E-3	2.97E-4	5.60E-5	5.70E-5	2.08E-4	3.74E-3	2.96E-4	4.42E-3

Table S5. *K values derived for each task and each subject. Table cells shaded in light blue indicate the lowest K value for a given task. Table cells shaded in yellow indicate the highest K value for a given task.*
